# The Role of a Community Surgeon in the Care of Hepatopancreatobiliary Patients: Short-Term Outcomes and Learning Curve

**DOI:** 10.7759/cureus.71388

**Published:** 2024-10-13

**Authors:** Elizabeth M Showalter, Ciarán T Bradley

**Affiliations:** 1 Surgical Oncology, Duly Health and Care, Downers Grove, USA

**Keywords:** community surgery, complex general surgical oncology, hepatopancreatobiliary surgery, learning curve, regionalization, volume-outcome relationship

## Abstract

Background

Owing to the well-established volume-outcome relationship, hepatopancreatobiliary (HPB) surgery is commonly regionalized to academic, teaching hospitals. However, regionalization is associated with decreased access for some populations in need, as well as geographic and financial barriers for patients. If high surgeon and institutional volumes can be achieved, the community, non-teaching HPB surgical practice could help alleviate some issues associated with regionalization. The HPB experience of a community surgeon immediately after surgical oncology training was reviewed, hypothesizing that high volumes with acceptable short-term outcomes could be achieved, although a learning curve may be observed.

Materials and methods

Electronic medical records from 2013 to 2023 were reviewed. Data included patient demographics, perioperative details, pathology, complications, and deaths over 90 postoperative days. Perioperative quality metrics were assessed for trends over time in pancreaticoduodenectomy (PD) and liver resection subgroups.

Results

A total of 295 patients underwent 176 (59.7%) pancreatic and 119 (40.3%) hepatobiliary operations. The most common operations were PD (n=87; 49.4%) and partial hepatic lobectomy (n=56; 41.1%). In the pancreas group, morbidity was 25% (n=44), and mortality was 4.5% (n=8). In the hepatobiliary group, morbidity and mortality were 19.3% (n=23) and 5.0% (n=6), respectively. Within the PD and liver resection subgroups, operative time, estimated blood loss, and hospital length of stay (LOS) trended downward over time, with LOS decreasing significantly.

Conclusion

High HPB volumes with acceptable short-term outcomes can be achieved by a solo practitioner in the community, non-teaching setting. For PDs and liver resections, perioperative metrics trended downward over time, illustrating the learning curve encountered after training.

## Introduction

There is a well-established association between surgeon and hospital volume and short- and long-term outcomes for hepatopancreatobiliary (HPB) operations, resulting in broad consensus for HPB operations to occur at high-volume centers [1-3]. However, controversy remains as to the optimal location and hospital setting in which to establish high-volume HPB centers [4-6]. While HPB surgery is often regionalized at predominantly urban, academic, and teaching hospitals, regionalization is associated with higher costs, increased disparities in access, and practical barriers such as geographical limitations, patient preferences, and payer requirements [7-10]. The community, non-teaching practice can potentially address these barriers to regionalization, provided high operative volumes are achieved.

Nevertheless, HPB operations are undeniably complex, technically demanding, and associated with a steep learning curve. The breadth of HPB training varies among three distinct fellowship pathways (complex general surgical oncology (CGSO), HPB surgery, and transplant surgery), and there is a predicted glut of HPB surgeons in the United States, clustered most notably around the coastal populations [11]. It is therefore debatable whether it is prudent to establish and maintain a community, non-teaching HPB practice in the face of such pressures, especially when larger, academic centers can ostensibly devote more personnel and resources to the care of these complicated patients.

## Materials and methods

We reviewed electronic medical records of consecutive patients who underwent liver, pancreas, and bile duct operations by the senior author (CTB) in the first 10 years of surgical oncology community practice after the CGSO fellowship (July 2013-July 2023). Duly Health and Care is an independent, suburban Chicago multispecialty group, of which the surgeon is the only CGSO, HPB, or transplant-trained surgeon. HPB surgery was a new service line. Operations occurred at several community hospitals, but by January 2019, they were consolidated exclusively to Northwestern Medicine Central DuPage Hospital (CDH), where the majority of HPB operations (90.2%) occurred. There were other, hospital-employed, CGSO-trained surgeons practicing at CDH. Cumulatively, the hospital performed an average of 45-50 pancreas resections and 20-25 liver resections annually.

Assistance in the operating room was variably provided by certified surgical assistants and physician assistants, and since 2018, almost exclusively by a single nurse practitioner (EMS). Other surgeons, with either general surgery, vascular surgery, or surgical oncology qualifications, were called upon as needed for occasional intraoperative assistance or counsel. The authors constituted the primary inpatient care team. Overnight and weekend cross-coverage was provided by general surgery colleagues from the same multispecialty practice. There was no resident participation.

We collected patient demographic and clinical data, perioperative quality metrics (operative time, estimated blood loss (EBL), hospital length of stay (LOS), rates and quantity of blood transfusion, microscopic negative resection margin (R0)), 90-day morbidity (Clavien-Dindo grade III or IV), and 90-day mortality [12]. The failure to rescue (FTR) rate was calculated as the percentage of deaths after major complications. Grades B and C postoperative pancreatic fistula (POPF) and delayed gastric emptying (DGE) were calculated according to the International Study Group of Pancreatic Surgery definitions [13,14]. We excluded open or minimally invasive pancreas or liver biopsies that were for diagnostic purposes only.

We employed descriptive statistics to compare rates of baseline characteristics between the pancreas and hepatobiliary groups. In pancreaticoduodenectomy (PD) and liver resection (open or minimally invasive) subgroups, we assessed for trends in the perioperative quality metrics using the Mann-Kendall Test for Trend. A p-value <0.05 was considered significant. Statistical analyses were conducted with XLSTAT (Lumivero, Denver, Colorado, United States). The study was exempt from Institutional Review Board approval per local protocol because all data were collected, tabulated, and stored in a de-identified manner. 

## Results

A total of 295 patients underwent 176 (59.7%) pancreatic operations and 119 (40.3%) hepatobiliary operations (Table 1). The 90-day morbidity and mortality in the pancreas group were 25% (n=44) and 4.5% (n=8), respectively. Within the hepatobiliary group, morbidity was 19.3% (n=23) and mortality was 5.0% (n=6). Failure to rescue for the entire cohort was 17.7%.

**Table 1:** Data related to hepatopancreatobiliary operations, 2013-2023 IQR: interquartile range; M/F: male/female; ASA: American Society of Anesthesiologists Physical Status

Characteristics	Total (n=295)	Pancreatic surgery (n=175)	Hepatobiliary surgery (n=119)
Age, median (IQR)	68.4 (16.4)	70 (13)	66.7 (21)
Gender, M/F (male%)	156/139 (53)	86/90 (45)	70/49 (59)
Race, n (%)			
White	235 (79.6)	145 (82.4)	90 (75.6)
Black	21 (7.1)	12 (6.8)	9 (7.6)
Hispanic	19 (6.4)	12 (6.8)	7 (5.9)
Other	20 (6.8)	7 (4.0)	13 (10.9)
ASA, n (%)			
1	3 (1.0)	0 (0)	3 (2.5)
2	76 (25.8)	44 (25)	32 (26.9)
3	201 (68.1)	122 (69.3)	70 (66.4)
4	15 (5.1)	12 (5.7)	5 (4.2)
Hospital length of stay (days), median (IQR)	6 (5)	6 (5)	5 (3.75)
90-day morbidity, n (%)	65 (22)	44 (25)	23 (19.3)
90-day mortality, n (%)	14 (4.7)	8 (4.5)	6 (5.0)
Failure to rescue, %	17.7	15.4	20.7

The most common pancreas operation was PD/total pancreatectomy (n=90; 51%), followed by minimally invasive distal pancreatectomy (n=45; 25.5%) and open distal or central pancreatectomy (n=35; 19.9%) (Table 2). Three patients underwent enucleation of a pancreas tumor (1.9%) and three underwent pancreatic necrosectomy (1.9%). The POPF rate was 13.3% (n=22). The rate of DGE was 4.0% (n=7). The causes of death after pancreas surgery were aspiration (n=2), portal vein thrombosis (n=1), POPF (n=1), cardiac arrest (n=1), acute respiratory failure (n=1), *Clostridioides difficile* colitis (n=1), and gram-negative septicemia after commencing adjuvant chemotherapy (n=1) (Table 3). 

**Table 2:** Complications and deaths by operation subtype * Traditional laparoscopic or robotic-assisted

Operation Subtype	n (%)	90-Day Morbidity, n (%)	90-Day Mortality, n (%)
Pancreatic Surgery	176	44 (25)	8 (4.5)
Pancreaticoduodenectomy/Total pancreatectomy	90 (51.1)	25 (27.8)	5 (5.6)
Minimally invasive distal pancreatectomy*	45 (25.5)	8 (17.8)	--
Open distal pancreatectomy/Central pancreatectomy	35 (19.9)	7 (20.0)	3 (8.8)
Pancreatic debridement/Necrosectomy	3 (1.7)	1 (33.3)	--
Enucleation of pancreas tumor	3 (1.7)	3 (100)	--
Hepatobiliary Surgery	119	23 (19.3)	6 (5.0)
Partial hepatic lobectomy	56 (47.1)	10 (17.9)	--
Hepatic lobectomy, Right	16 (13.4)	5 (31)	3 (18.8)
Hepatic lobectomy, Left	8 (6.7)	2 (25)	--
Trisegmentectomy	5 (4.2)	1 (20)	1 (20)
Minimally invasive liver resection for solid tumor*	18 (15.1)	3 (16.7)	1 (5.6)
Laparoscopic fenestration/Drainage of cyst/Abscess	11 (9.2)	1 (9)	--
Bile duct resection and/or Bilioenteric anastomosis	3 (2.5)	--	1 (33.3)
Open microwave ablation	1 (0.8)	--	--
Open abscess drainage	1 (0.8)	1 (100)	--

**Table 3:** Causes of complications and deaths according to type of surgery ^a ^Complications in each group do not add up to the total, because a single patient can experience more than one complication Data given as frequency except where marked as n (%) GI: gastrointestinal; CVA: cerebrovascular accident; VTE: venous thromboembolism; MSOF: multiple systems organ failure

Causes	Pancreatic (n=176)	Hepatobiliary (n=119)
90-Day Morbidity, n (%)	44 (25)^a^	23 (19.3)^a^
Pancreatic fistula	22	N/A
Bile leak	4	8
Abscess/Fluid collection/Infection	6	8
Delayed gastric emptying	7	N/A
Hemorrhage or GI bleeding	7	4
GI perforation or leak	4	2
Jaundice/Biliary stricture	3	2
Pleural effusion	2	1
Respiratory failure	--	2
Esophagitis	1	--
CVA	1	--
VTE	1	--
Liver failure	--	1
Aspiration	1	--
Clogged feeding tube	1	--
90-Day Mortality, n (%)	8 (4.5)	6 (5.0)
Portal vein thrombosis-intestinal ischemia	1	--
Pancreatic fistula	1	--
Liver failure	--	2
Septic shock – MSOF	--	2
Hemorrhage	--	1
Aspiration	2	1
Cardiac arrest	1	--
*Clostridioides difficile *colitis	1	--
Respiratory Failure	1	--
Gram-negative septicemia on adjuvant chemotherapy	1	--

The most common hepatobiliary operation was partial hepatic lobectomy (n=56; 47.1%), followed by minimally invasive liver resection (n=18; 15.1%), right hepatic lobectomy (n=16; 13.4%), minimally invasive cyst fenestration or abscess drainage (n=11; 9.2%), left hepatic lobectomy (n=8; 6.7%), trisegmentectomy (n=5; 4.2%), and bile duct resection and/or bilioenteric anastomosis (n=3; 2.5%). One patient each underwent open microwave ablation (0.8%) and open abscess drainage (0.8%). The causes of death after hepatobiliary surgery were liver failure (n=2), septic shock associated with multiple systems organ failure (n=2), hemorrhage (n=1), and aspiration (n=1). 

The four most common histologies in the pancreas group (10 operations) in descending order were pancreatic ductal adenocarcinoma, primary pancreatic neuroendocrine tumor, intraductal papillary mucinous neoplasm, and carcinoma of the Ampulla of Vater (Table 4). The five most common histologies in the hepatobiliary group (10 operations) were metastatic colorectal cancer, hepatocellular carcinoma, simple hepatic cyst, intrahepatic cholangiocarcinoma, and hepatic adenoma. In both groups, approximately 21% of operations were for less common histologies and reported as “other”.

**Table 4:** Hepatopancreatobiliary operations by underlying pathology

Pancreas (n=176)	Frequency (Percentage)
Pancreatic ductal adenocarcinoma	66 (37.5)
Pancreatic neuroendocrine tumor	30 (17.0)
Intraductal papillary mucinous neoplasm	29 (16.5)
Ampullary cancer	14 (7.8)
Other	37 (21)
Hepatobiliary (n=119)	
Metastatic colorectal cancer	42 (35.3)
Hepatocellular carcinoma	18 (15.1)
Cyst	12 (10.1)
Intrahepatic cholangiocarcinoma	11 (9.2)
Adenoma	10 (8.4)
Other	26 (21.8)

**Figure 1:**
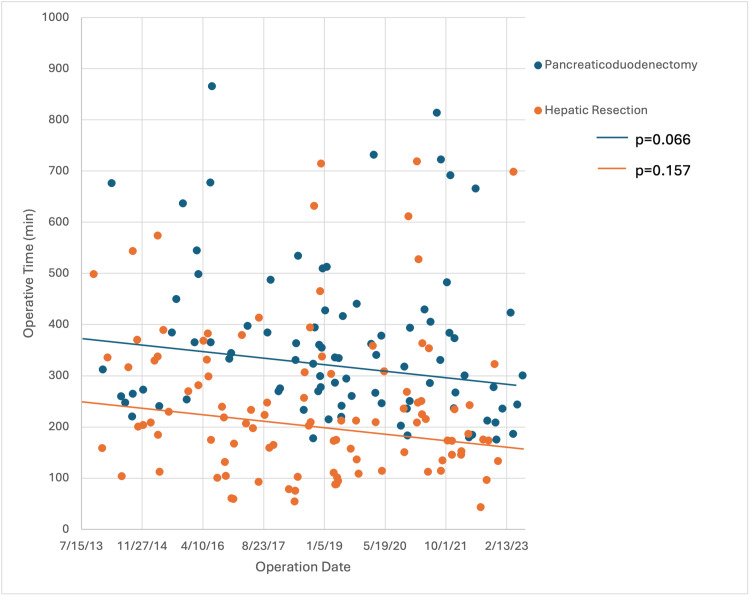
Operative time of pancreaticoduodenectomies and liver resections over the study period

**Figure 2:**
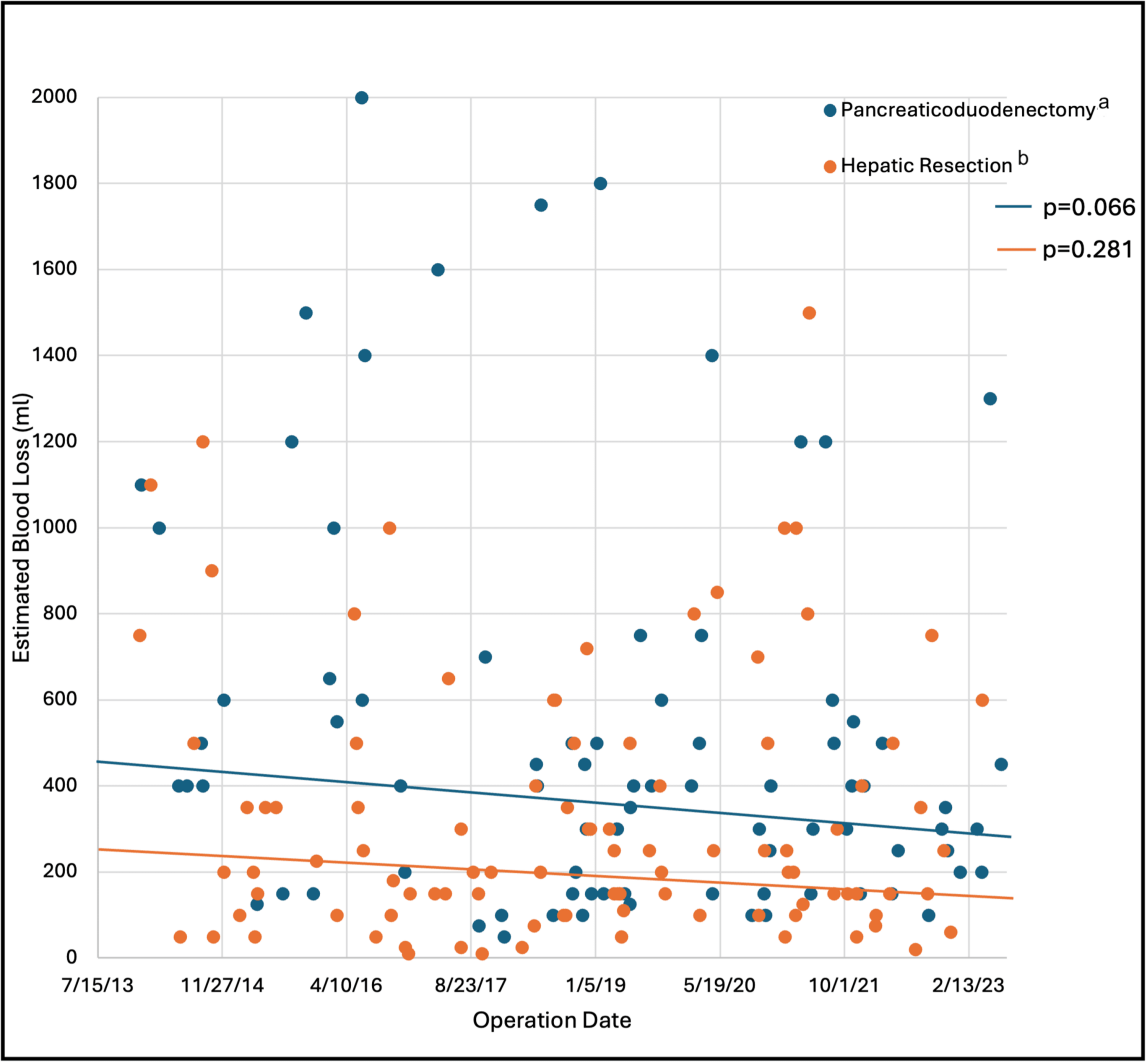
Estimated blood loss of pancreaticoduodenectomies and liver resections over the study period ^a^Two pancreaticoduodenectomies with EBL 12,000 ml and 5,000 ml that are included in the analysis are not depicted on the graph because the scale of the Y-axis could not accommodate. ^b^Five liver resections with EBL 19,000 ml, 13,000 ml, 4,000 ml, 2,500 ml, and 2,400 ml that are included in the analysis are not depicted on the graph because the scale of the Y-axis could not accommodate.

**Figure 3:**
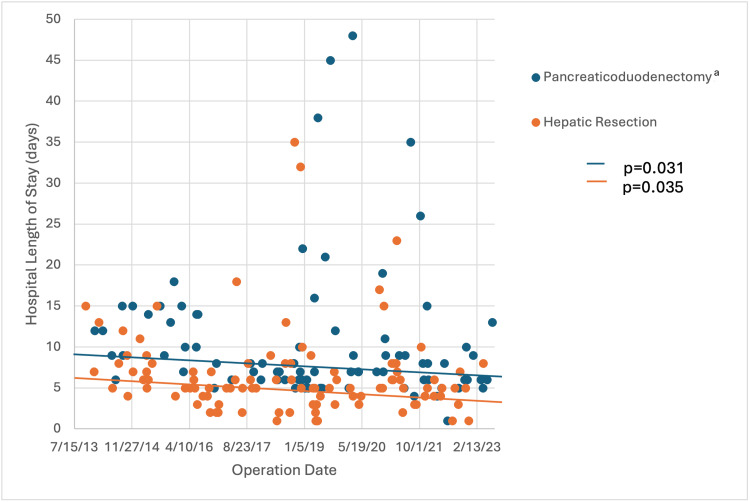
Hospital length of stay for pancreaticoduodenectomies and liver resections over the study period ^a^Two pancreaticoduodenectomies with hospital length of stays of 83 days and 56 days that are included in the analysis are not depicted on the graph because the scale of the Y-axis could not accommodate

Regarding the learning curve for PD (n=87) and liver resection (n=103) subgroups, trends in perioperative quality metrics over the 10-year study period are presented in Table 5. While broad ranges were noted, operative time (Figure 1), EBL (Figure 2), and LOS (Figure 3) had decreasing trend slopes, but only LOS decreased significantly, after both PD and liver resection. Nineteen PD patients (21.8%) and 12 liver resection patients (11.7%) required intraoperative transfusions. When blood was administered, the number of packed red blood cells transfused during PD (median, 3 units) and liver resection (median, 2 units) was stable over the study period. R0 resection rate in both subgroups was similar: 94.0% during PD and 94.2% during liver resection. 

**Table 5:** Trends in perioperative quality metrics over the study period for pancreaticoduodenectomies and liver resections IQR: interquartile range; PRBCs: packed red blood cells; R0: microscopically negative margin ^a^ Three pancreaticoduodenectomies for non-neoplastic conditions excluded from the denominator; ^b^ Categorical variables limited to descriptive statistics only Data given as: *median (IQR), (min-max); **n (%)

	Operative Time (minutes)	EBL (ml)	Hospital Length of Stay (days)	Intraoperative Transfusion^b^	Units of PRBCs per Transfusion	R0 Resection^b^
Pancreaticoduodenectomy (n=87)	331 (189), (176-866)*	400 (400), (50-12,000)*	8 (6.5), (4-83)*	19 (21.8)**	3 (2), (1-17)*	79^a^ (94)**
Sen’s Slope	-0.025	-0.038	-0.001	N/A	0.000	N/A
p-value	0.066	0.226	0.031	N/A	0.542	N/A
Liver Resection (n=103)	210 (178.5), (44-719)*	225 (275), (10-19,000)*	5 (3), (1-35)*	12 (11.7)**	2 (4.25), (1-27)*	97 (94.2)**
Sen’s Slope	-0.019	-0.022	-0.001	N/A	-0.001	N/A
p-value	0.157	0.281	0.035	N/A	0.105	N/A

## Discussion

The volume-outcome relationship is well established in HPB surgery, leading many to offer minimal threshold recommendations for surgeons and hospitals. The most cited volume recommendation in pancreas surgery is that from the Leapfrog Group. They recommend greater than 10 pancreas resections for cancer per surgeon and greater than 20 operations per hospital per year. No such recommendations exist for hepatobiliary surgery, but authors have variably classified high-volume liver surgery centers as those that perform greater than three to greater than 45 liver resections per year [4,6,7,15-20]. A suggested individual surgeon volume threshold for liver surgery is more elusive still, although Scarborough et al. stratified a cohort of liver resection patients from the National (Nationwide) Inpatient Sample from 1998 to 2003 into quintiles, where the highest volume surgeons performed greater than 16 liver resections annually. Those who performed 6-16 liver resections were classified as high-intermediate volume [10]. 

Here, we presented the largest series of HPB operations within the surgical literature performed by a single surgeon in a community-based, non-teaching, non-academic practice. Ehnstrom and colleagues published the HPB experience of a single surgeon at a non-teaching hospital in Hawai’i [21]. They reported outcomes on 92 pancreas operations and 71 hepatobiliary operations over 14 years, constituting what they classified as “low volume”. 

Our high-volume, single-surgeon experience (average 17 pancreas and 11 hepatobiliary operations annually) occurred in the context of a high-volume hospital, where there are other CGSO-trained surgeons performing HPB operations. We believe this is key to a successful community-based HPB practice because although these surgeons belong to a different medical practice, and there is limited interaction or collaboration between the two groups, there are shared resources that benefit all, including specialized anesthesia, operating room, and nursing teams. We have noted continued process improvements and staff familiarity with these complex operations. 

Our morbidity and mortality are modestly high compared to those reported by academic centers but remain within the range of national database reviews, where 90-day mortality after PD approaches 8.2% and in-hospital mortality after liver resection is 4.7% [10,22-24]. It is both humbling and promising to note that within our pancreas group, the postoperative deaths were clustered in the earlier stages of our experience and may be diminishing. Seven deaths occurred within the first six years (101 operations) while only one death was observed within the last four years (75 operations). Our FTR rate was 15.4% in the pancreas group and 20.7% in the hepatobiliary group, which are moderately high and can help inform additional systematic and quality improvements within our practice and hospital.

The push for regionalization of HPB care stems from the laudable goal of ensuring HPB operations occur at high-volume centers where patient outcomes are better. However, regionalization has unintended consequences, such as creating disparities in access for older patients with more comorbidities, minorities, and patients from low-income communities [10]. Moreover, patients report long travel times and travel costs as barriers to seeking care at high-volume centers, and few patients are even aware of the volume-outcome relationship [25]. Our experience demonstrates that, provided high individual surgeon and high overall hospital HPB volumes are ensured, HPB care can be “regionalized in the community”. These findings have policy implications for healthcare systems and payors seeking to promote quality, contain costs, and situate specialized care close to communities in need.

The learning curve in HPB surgery is not insignificant. Fifteen percent of surveyed CGSO graduates feel clinically unprepared for managing HPB diseases, while 24% of the same cohort report being technically unprepared to perform HPB operations [26]. Several authors have demonstrated improved perioperative outcomes after increased numbers of pancreas operations performed. Tseng et al. examined EBL, operative time, LOS, and margin status for three high-volume, academic surgeons performing PD [27]. They selected a breakpoint of 60 cases after which outcomes improved. This pattern continued up to 360 PDs when examined for one particular surgeon. Hardacre observed a similar phenomenon when comparing his first 30 PDs immediately after training to his second 30 [28]. In his experience, operative time and LOS decreased, but morbidity, mortality, and margin status remained the same. Finally, in likely the largest published, single-surgeon experience of 2000 consecutive PDs, Cameron and He reported decreased operative time, units of packed red blood cells transfused, and LOS by decade from the 1980s to 1990s and then 2000s [29]. They observed stable rates of POPF and DGE over their study period. 

We believe the post-training learning curve for HPB surgery is steepest in the first few years of practice, particularly when entering a non-academic, non-teaching practice as a solitary HPB surgeon. We chose to examine our perioperative metrics in the subgroups of PD and liver resections to achieve some element of homogeneity. Our analysis was novel insofar as we treated time in practice as the continuous, independent variable. We postulate that a surgeon’s efficiency and safety when performing a particular operation such as PD is influenced not just by the previous number of PDs performed, but by any number of other anatomically or technically similar operations performed over time that might be for diseases unrelated to the pancreas or liver. For instance, the delicate dissection of bulky metastatic nodal disease at the base of the small bowel mesentery from a small intestinal neuroendocrine tumor is not markedly different from the dissection of advanced pancreatic adenocarcinoma of the uncinate along the distal superior mesenteric vein and artery. Cumulative time in practice accounts for the other operations that occur in between HPB operations that help advance one’s technical skill and intraoperative decision-making. Be that as it may, in a recent national review of 11,746 cancer operations among Medicare beneficiaries performed by 676 CGSO graduates, surgeon operation-specific volume was associated with lower odds of serious complications, lending further credence to the contrasting argument that accrued experience with one specific operation may be the key to mastering the learning curve [30]. 

Our analysis demonstrated negatively sloped trends in operative time, EBL, and LOS over the 10-year study period for both PDs and liver resections. However, only LOS decreased significantly in both subgroups. We surmise that the downward trend in operative time and EBL didn’t reach statistical significance because of the undue influence of prominent outliers. Consistency and reproducibility can and should improve over time. 

This study has several limitations. Firstly, by nature of the retrospective design, it is subject to bias. We now have the mechanics in place to prospectively maintain our HPB database, which will help reduce, but not eliminate, bias in the future. Secondly, as they represent the experience of a single surgeon servicing a particular referral base in a suburban setting, the data are questionably generalizable. Likewise, while our cohort is larger than other community-based HPB experiences, the overall sample size is still relatively small. Rates of typically rare events, such as mortality, are most affected by a small denominator. Rare diseases (e.g. hilar cholangiocarcinoma) and unusual operations (e.g. extrahepatic bile duct resection) are underrepresented. We look forward to the analysis afforded by the next 10 years of HPB practice. Thirdly, our subgroup analysis of perioperative quality metrics is limited by significant heterogeneity, because not all PDs or liver resections are the same. Within the PD subgroup, operations that required a venous resection/reconstruction or multivisceral resection were grouped together with the “textbook” cases. Likewise, any liver resections of one segment or more, whether laparoscopic or open, were analyzed as one group, even though one can reasonably assume that metrics such as operative time and EBL increase proportionately with the volume of liver to be resected. Minimally invasive operations are also subject to heavy selection bias. 

## Conclusions

While most high-volume HPB surgeons will continue to operate at high-volume teaching facilities, there may continue to be a role for community-based HPB surgery, which can help address barriers created by regionalization of care. Hgh individual surgeon HPB operative volume with acceptable short-term outcomes can be achieved in a community, non-teaching practice. To address a perceived learning curve, which is likely most pronounced in the first years after training, HPB surgeons in the community setting should pool institutional resources to establish high HPB institutional volumes, while supporting each other despite practice barriers. 
